# Outcome of transcatheter aortic valve replacement in patients over 85 years of age versus patients aged 85 and younger

**DOI:** 10.1007/s12471-022-01693-9

**Published:** 2022-05-24

**Authors:** F. S. van den Brink, I. Wijtsma, H. Amrane, T. N. E. Vossenberg, J. Haenen, F. Porta, A. J. Van Boven, S. H. Hofma

**Affiliations:** 1 Department of Cardiology, Medical Centre Leeuwarden (MCL), Leeuwarden, The Netherlands; 2 Department of Anaesthesiology, Medical Centre Leeuwarden (MCL), Leeuwarden, The Netherlands; 3 Department of Cardio-thoracic surgery, Medical Centre Leeuwarden (MCL), Leeuwarden, The Netherlands

**Keywords:** Transcatheter aortic valve replacement, Outcome, Octogenarians

## Abstract

**Introduction:**

The Dutch general population is aging rapidly. Many of these patient are fit and eligible for TAVR. However, studies on outcome in older versus younger patients are scant.

**Material and methods:**

A single-centre retrospective study comparing patients older and younger than age 85 on outcome.

**Results:**

190 patients underwent TAVR: 136 were aged 85 or younger (U85), 54 were older than 85 (O85). The U85 group had more men (U85: 71 [52.2%] vs O85: 19 [35.2%]; *p* = 0.034), a higher incidence of diabetes (U85: 36 [26.5%] vs O85: 3 [5.6%]; *p* = 0.001) and atrial fibrillation (U85: 35 [25.7%] vs O85: 5 [9.3%]; *p* = 0.03) and a higher body mass index (U85: 27.5 [± 5.24] vs O85: 26 [± 3.78]; *p* = 0.027). In the O85 group there was a lower estimated glomerular filtration rate (O85: 50.28 [± 15.32] ml/min vs U85: 65.25 [± 29.97] ml/min; *p* = 0.012). There was no difference in 30-day mortality (U85: 6 [4.4%] vs O85: 3 [5.6%]) and 1‑year mortality (U85 9 [6.6%] vs O85 3 [5.6%]) (*p* = 0.521). There was an equal amount of new onset permanent left bundle branch block (U85: 38 [27.9%] vs O85: 14 [25.9%]; *p* = 0.896) and permanent pacemaker implantation (U85: 28 [20.6%] vs O85: 28 [20.6%]; *p* = 0.748). There was no difference in bleeding events (*p* = 0.469), vascular complications (*p* = 0.195) or moderate/severe regurgitation (*p* = 0.972). The U85 group had a slightly longer admission duration (U85 6.29 [± 5.289] days vs O85 5.98 [± 3.328] days (*p* = 0.037)).

**Conclusion:**

TAVR in patients over 85 years of age has excellent outcome, comparable to those aged 85 and younger.

## What’s new?

- In this single-centre study, transcatheter aortic valve replacement has excellent short-term and intermediate-term outcome in octogenarians and nonagenarians over 85 years of age.
- In this single-centre observational study, outcome in patients over 85 years of age is just as good as in patient aged 85 and younger.
- Patients undergoing transcatheter aortic valve replacement under 85 years of age tend to have higher incidences of diabetes and atrial fibrillation, and a higher body mass index, while those over 85 years of age have poorer renal function.

## Introduction

The most common valvular disease in the western world is aortic valve stenosis, which rises in incidence with increasing age. Approximately 10% of people of age 80 and older suffer from this degenerative disease [1–3]. Untreated it leads to left ventricular failure and eventually death [4–6]. As no effective medical therapy is known, the only treatment available is replacement of the affected valve [7, 8]. Historically, this was done by surgically replacing the diseased valve with a prosthetic aortic valve (SAVR). However, about one-third of patients with aortic valve stenosis was deemed unfit for SAVR [9]. Since 2002, an alternative treatment for these patients is available in the form of transcatheter aortic valve replacement (TAVR) [10]. Initially, this technique was only used in so-called “high-risk patients” who were unfit to undergo SAVR, but as time progressed intermediate risk and low-risk patients also became eligible for TAVR [11–16]. At the same time, there is an increase in patients over 85 years of age due to an aging population with an estimated 187,000 people over 90 years of age in the Netherlands in 2030 according to the Dutch Central Bureau of Statistics. These patients are prone to developing aortic valve stenosis which may be an indication for treatment. This study was performed to assess the outcome of TAVR in patients over age 85 compared with patients aged 85 and younger in regard to mortality and morbidity.

## Material and methods

A single-centre retrospective study was performed including all patients undergoing a TAVR at the Medical Centre Leeuwarden (MCL), Leeuwarden, the Netherlands, from January 2017 until November 2019. Data were extracted from the database of the Netherlands Heart Registry (NHR) [17]. All patients underwent evaluation by the Heart Team prior to TAVR. Prior to the Heart Team’s evaluation all patients underwent coronary angiography, aortic computed tomography, transthoracic and—on indication—transoesophageal echocardiography, spirometry and assessment of frailty and vitality in either an outpatient clinic setting or in a clinical setting when admitted to hospital [18].

For this study patients were analysed at baseline for age, gender, previous coronary and valvular interventions, diabetes, neurological status, pulmonary disease, New York Heart Association (NYHA) Class, left ventricular ejection fraction (LVEF), history of atrial fibrillation, creatinine levels (μmol/l), body mass index (BMI), aortic valve area (AVA in cm^2^), estimated glomerular filtration rate (eGFR in ml/min) and European System for Cardiac Operative Risk Evaluation (EuroSCORE) II. The two age groups were defined as all patients aged 85 and younger (U85) and all patients aged 86 and older (O85).

Procedural characteristics were analysed for access site, valve type, valve size in mm and use of pre-dilatation and post-dilatation.

Outcome was analysed in regard to 30-day mortality, 1‑year mortality, post-implantation aortic regurgitation and paravalvular leakage, onset of new left bundle branch block (LBBB), new pacemaker device implantation, post-procedural, bleeding complications, vascular complications, readmission within 30 days, post-procedural pericardial tamponade, neurological complications including post-procedural delirium and acute kidney injury. Definitions of these complications were in accordance with the Valve Academic Research Consortium 2 (VARC-2) [19–21].

LVEF was defined as normal (LVEF > 50%), mildly (LVEF 30–49%), moderately (20–29%) or severely impaired (LVEF < 20%). Outcomes are given as absolute numbers with the percentages in brackets.

Statistical analysis was performed using the Kolmogorov-Smirnov test to assess normality for the continues variables. If there was normal distribution, Student’s t‑test was performed. If distribution was not normal, we performed a Mann-Whitney U test to assess significance. Categorial variables were analysed using a chi-squared test. Survival after 1 year was assessed using a non-parametric Kaplan-Meier test. A log-rank test was performed to assess a significant difference between the two. Statistical analysis was performed using SPSS (IBM, Armonk, USA). A two-tailed *p*-value of < 0.05 was considered statistically significant. In accordance with the Medical Research Involving Human Subjects Act (WMO), a medical ethics committee declared there was no ethical conflict with regard to this study.

## Results

### Baseline characteristics

A total of 190 patients underwent TAVR between January 2017 and November 2019. A total of 136 patients was 85 years or younger (U85) and there were 54 patients who were over 85 years of age (O85). The median age was 78.5 (± 5.5; range 55–85) years and 87.3 (± 2.95; range 86–98) years (*p* = 0.001) respectively. In the U85 group there were significantly more men (71, 52.2%) than in O85 (19, 35.2%) (*p* = 0.034). A significantly larger number of patients in U85 had diabetes (36 [26.5%] vs 3 [5.6%] in O85 (*p* = 0.001)) and a significantly larger number of patients had a history of atrial fibrillation (U85 35 [25.7%] vs O85 5 [9.3%]; *p* = 0.03). The patients in the U85 group also had a slightly but significantly higher BMI (27.5 [± 5.24] vs 26 [± 3.78]; *p* = 0.027). In the O85 group there was a higher number of patients with decreased eGFR (O85 50.28 [± 15.32] ml/min vs U85 65.25 [± 29.97] ml/min; *p* = 0.012).

The majority of patients were in NYHA Class 3–4 in both groups and there was no difference in the EuroSCORE II or AVA between both groups. In both groups, the majority of TAVR procedures were performed in an elective setting (U85 99 [72.8%] vs O85 42 [77.8%]; *p* = 0.48), where the patient was scheduled in advance. The remaining patients were admitted with heart failure and underwent TAVR implantation after re-compensation in a clinical setting.

Otherwise, there were no statistically significant differences between the two groups (see Tab. 1).

**Table 1:** Baseline characteristics

	Age ≤ 85 years (*N* = 136)	Age > 85 years (*N* = 54)	*P*-value
*Age (years)*	78.5 ± 5.50	87.33 ± 2.95	0.001
*Man*	71 (52.2%)	19 (35.2%)	0.34
*Diabetes*	36 (26.5%)	3 (5.6%)	0.001
*TIA/CVA*	32 (23.5%)	9 (16.7%)	0.32
*Neurological dysfunction*	10 (7.4%)	1 (1.9%)	0.14
*COPD*	30 (22.1%)	8 (14.8%)	0.26
*PCI*	37 (27.2%)	10 (18.5%)	0.21
*CABG*	30 (22.1%)	8 (14.8%)	0.26
*Prior aortic valve surgery*	14 (10.3%)	2 (3.7%)	0.14
*NYHA 3–4*	111 (81.6%)	47 (87%)	0.37
*Elective procedure*	99 (72.8%)	42 (77.8%)	0.48
*LVEF >* *50%*	95 (69.9%)	40 (74.1%)	0.56
*LVEF 30–49%*	32 (23.5%)	11 (20.4%)	0.64
*LVEF 20–29%*	9 (6.6%)	3 (5.6%)	0.79
*LVEF <* *20%*	0	0	
*Pre-existent AF*	35 (25.7%)	5 (9.3%)	0.03
*Creatinine*	95.2 ± 31.4	90.2 ± 27.8	0.825
*BMI*	27.5 ± 5.24	26 ± 3.78	0.027
*AVA in cm*^*2*^	0.79 ± 0.22	0.73 ± 0.18	0.373
*eGFR*	65.25 ± 29.97	50.28 ± 15.32	0.012
*EuroSCORE II*	6.00 ± 8.17	6.76 ± 6.28	0.764

*TIA* transient ischaemic attack, *CVA* cerebrovascular accident, *COPD* chronic obstructive pulmonary disease, *PCI* percutaneous coronary interventions, *CABG* coronary artery bypass graft, *NYHA* New York Heart Association, *LVEF* left ventricular ejection fraction, *AF* atrial fibrillation, *BMI* body mass index, *AVA* aortic valve area, *eGFR* estimated glomerular filtration rate

### Procedural characteristics

In both groups, the preferred access site was transfemoral (U85 114 [83.8%] vs O85 [83.3%]) with a smaller amount of patients who underwent a direct aortic approach TAVR (U85 21 [15.4%] vs O85 8 [14.8%]). In U85, one patient (0.7%) underwent a TAVR via transapical approach and in O85 one patient underwent TAVR via a subclavian approach (*p* = 0.507, Tab. 2).

**Table 2:** Procedural characteristics

	Age ≤ 85 years *N* = 136	Age > 85 years *N* = 54	Total *N* = 190	*P*-value
*Access site*				
Femoral	114 (83.8%)	45 (83.3%)	159	
Direct aortic	21 (15.4%)	8 (14.8%)	29	
Subclavian	0	1 (1.9%)	1	
Apical	1 (0.7%)	0	1	0.507
*Valve type*				
Edwards	15 (11%)	3 (5.6%)	18	
Medtronic	75 (55.1%)	32 (59.3%)	107	
StJude	42 (30.9%)	19 (35.2%)	61	
Boston	4 (2.2%)	0	4	0.533
*Valve size*				
20–26 mm	31 (23%)	17 (31.5%)	48	
27–34 mm	105 (77%)	37 (68.5%)	141	0.214
*Pre-dilatation*				
No	83 (61%)	30 (55.6%)	113	
Yes	53 (39%)	24 (44.4%)	77	0.488
*Post-dilatation*				
No	96 (70.6%)	38 (70.4%)	134	
Yes	40 (29.4%)	16 (29.6%)	56	0.976

The majority of patients received a self-expandable valve of either Medtronic, Inc. (Minneapolis, Minnesota, USA), St Jude Medical Inc. (St. Paul, Minnesota, USA) or Boston Scientific Corporation (Marlborough, Massachusetts, USA). A smaller amount of patients received a balloon expandable valve of Edwards Lifesciences Corporation (Irvine, California, USA). There was no significant difference in the type of implanted valves between the two groups (*p* = 0.533), nor in valve size (*p* = 0.214), nor in amount, nor in pre-dilatation (*p* = 0.448) and post-dilatation (*p* = 0.976) (see Tab. 2).

### Outcome

**Fig. 1:**
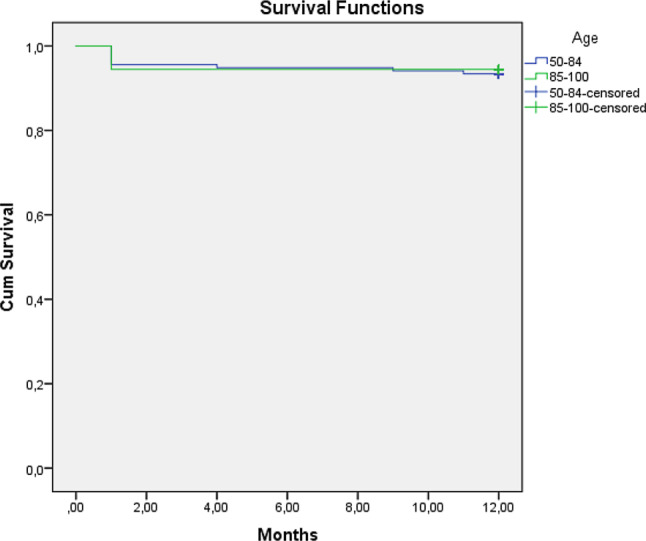
*Kaplan-Meier analysis and curve.* 1‑year survival of patients aged ≤ 85 and > 85 (*p* = 0.791)

There was no peri-procedural mortality in either group. There was no difference in either 30-day mortality (U85 6 [4.4%] vs O85 3 [5.6%]) or 1‑year mortality (U85 9 [6.6%] vs O85 3 [5.6%]) between the two groups (*p* = 0.521). See Tab. 3 and Fig. 1.

**Table 3:** Outcome

	Age ≤ 85 years *N* = 136	Age > 85 years *N* = 54	Total *N* = 190	*P*-value
*Post-operative AR/PVL*				
None	45 (33.3%)	18 (33.3%)	63	
Grade 1	65 (48.1%)	27 (50%)	92	
Grade 2	21 (14.5%)	7 (13%)	28	
Grade 3	3 (2.2%)	2 (2.7%)	5	
Grade 4	2 (1.5%)	0	2	0.972
*Post-operative LBBB*				
None	87 (64%)	34 (63%)	121	
Temporary	11 (8.1%)	5 (9.3%)	16	
Upon Discharge	38 (27.9%)	14 (25.9%)	52	0.896
*Permanent pacemaker implantation*				
None	108 (79.4%)	44 (81.5%)	152	
PM	28 (20.6%)	10 (18.5%)	38	0.748
*Bleeding complications*				
None	126 (92.6%)	48 (88.9%)	174	
Type 1	2 (1.5%)	3 (1.6%)	5	
Type 2	3 (2.2%)	1 (1.9%)	4	
Type 3	5 (3.7%)	2 (3.7%)	7	0.469
*Vascular complications*				
None	125 (91.9%)	45 (83.3%)	170	
Minor	6 (4.4%)	4 (7.4%)	10	
Major	5 (3.7%)	5 (9.3%)	10	0.195
*Admission length (days)*	6.29 ± 5.289	5.98 ± 3.328		0.037
*Readmission within 30 days*				
None	124 (91.2%)	50 (92.6%)	174	
Admission	12 (8.8%)	4 (7.4%)	16	0.751
*Tamponade*				
None	135 (99.3%)	53 (98.1%)	188	
Tamponade	1 (0.7%)	1 (1.9%)	2	0.232
*TIA/CVA/delirium*				
None	124 (91.2%)	49 (90.7%)	173	
TIA	0	1 (1.9%)	1	
CVA	5 (3.7%)	0	5	
Delirium	7 (5.1%)	4 (7.4%)	11	0.924
*Acute kidney injury*				
None	131 (96.3%)	54 (100%)	185	
AKI	5 (3.7%)	0	5	0.153
*Death*				
None	127 (93.4%)	51 (94.9%)	178	
30 days	6 (4.4%)	3 (5.6%)	9	
1 year	9 (6.6%)	3 (5.6%)	12	0.521

*AR* aortic regurgitation, *PVL* paravalvular leakage, *LBBB* left bundle branch block, *PM* pacemaker, *TIA* transient ischaemic attack, *CVA* cerebrovascular accident, *AKI* acute kidney injury

The number of patients with mild residual aortic regurgitation or paravalvular leakage was comparable in both groups. Only a small group of patients had moderate regurgitation (U85 5 [3.7%] vs O85 2 [2.7%]) and this was not statistically different between the groups (*p* = 0.972, see Tab. 3).

Both groups had equal incidence of new onset LBBB, both temporary and upon discharge (*p* = 0.896). The need for permanent pacemaker device implantation was equal in both groups as well (*p* = 0.748). Vascular and bleeding complications occurred in only a small group of patients in both groups. Neither of these complications differed significantly between the two groups with regard to bleeding (*p* = 0.469) and vascular complications (*p* = 0.195). When considering neurological complications there was also no difference between the two groups (*p* = 0.924). With regard to readmission within 30 days (*p* = 0.751), post-procedural tamponade (*p* = 0.232) and acute kidney injury (*p* = 0.153) both groups performed equally well (Tab. 3: Outcome).

The only difference in outcome between the groups was that the U85 group had a slightly longer admission duration after TAVR (U85 6.29 [± 5.289] days vs O85 5.98 [± 3.328] days; *p* = 0.037) (Tab. 3: Outcome).

Another finding in this study is that patients undergoing a direct aortic approach in both groups combined have a much worse 1‑year survival than patients who had a transfemoral approach (transfemoral mortality 7 [4.4%] vs direct aortic mortality 5 [17.2%]; *p* = 0.0093) (Tab. 3: Outcome).

## Discussion

This study found that patients over 85 years of age have a similar outcome as patients aged 85 and younger when undergoing TAVR. This is in line with two other studies with patients of advanced age undergoing TAVR performed by Havanuk et al. and Vendrik et al. [22, 23]. A possible explanation for this fact is that patients in the O85 group are healthier and fitter than their younger counterparts. In this group there is a lower incidence of diabetes and atrial fibrillation. O85 patients are also less frequently overweight. Despite the fact that they have poorer kidney function, they still perform very well and have no post-procedural acute kidney injury. Although no hard evidence is provided by this study, we believe this underlines the role of the Heart Team in selecting the patients of over 85 years of age for TAVR and thus ensuring good short-term and intermediate-term prognosis. However, this is also one of the major limitations of this study as there is a highly selected population of patients of over 85 years of age.

The fact that there are more women in the O85 group is explained by the fact that women have a higher life expectancy than men as demonstrated in the latest report from the United Nations [31]*.*

The high number of patients that die within one year in the direct aortic approach group is in line with previously published literature [24]. Patients who need the direct aortic approach generally have much poorer vasculature. This is a symptom of extensive atherosclerosis, which in itself is a result of poorer overall health. Due to the very low number of patients undergoing either transaxillary or transapical TAVR it is not possible to draw any sound conclusions in this respect.

The relatively high number of pacemaker implantations was higher than in a previous study performed in the Netherlands [23]. This may be explained by the fact that a relatively larger number of patients is this study received a self-expandable prosthetic aortic valve, and by the large sizes of valves used. The self-expandable prosthetic aortic valve performs just as well as the balloon-expandable prosthetic heart valve, except for conduction disorders and the need for permanent pacemaker device implantation. A pre-existing LBBB or right bundle branch block predisposes for the need for permanent pacemaker device implantation [25, 26]. In the current era this is anticipated by the Heart Team and in case of very high pacemaker risk, for example first-degree AV block and wide QRS complex right bundle branch block, prior pacemaker implantation is recommended. However, this approach was adopted in 2019 and not yet implemented in most of the patients from the cohort of this study.

Although statistically not significant, the percentage of patients over 85 years of age experiencing vascular complications is almost threefold (O85 9.3% vs U85 3.7%). A possible explanation for this is that older patients have poorer vasculature, and are therefore prone to complications. Also operator experience with closure devices such as the Manta closure device (Teleflex Inc. Wayne, Pennsylvania, USA) is pivotal in preventing these complications.

A shortcoming of this study is that it has only complete data available on 30-day and 1‑year mortality. In this study, there were no data available on improvement on the quality of life (QOL). Although current practice does include filling in QOL forms, data in this regard were incomplete for this study, and therefore not included. Data from the Netherlands Heart Registry (NHR) [32]*,* a national mandatory registry for centres that perform cardiovascular interventions, demonstrated that patients aged 85 and younger not only improve in physical, but also in psychological well-being. Patients over 85 years of age only improve in physical well-being. Future studies should confirm this.

## Limitations

Whether or not TAVR is a cost-effective treatment is a question that this study does not answer. The answer becomes increasingly relevant with increasing age. Aortic valve stenosis is associated with high mortality and morbidity [5, 6]. Untreated, it leads to frequent hospital admissions [27, 28]. So, to answer the question of whether or not TAVR increases the quality-adjusted life years in the elderly, a cost analysis must be made comparing the costs of the TAVR treatment with those of conservative treatment with frequent readmissions. This is pivotal in assuring reimbursement for this costly therapy. Another important consideration is whether the valve replacement allows patients to remain self-supporting and can prevent admission to a nursing home with concomitant costs.

This study also does not address the frailty of patients undergoing TAVR. Frailty is an important risk factor for hospitalisation, morbidity and mortality [29]. Data in this regard was incomplete and therefore not included in this study. However, other studies, such as the one performed by Anand et al., demonstrated a relation between frailty and outcome [30].

There are various other limitations to this study and its design. It is a retrospective study with all the shortcomings of such a study. There is huge selection bias in the patient population, and the study did not evaluate the outcome in patients who had been turned down for intervention. Although there is causality in the results, no relation has been established.

## Conclusion

TAVR in selected patients of over 85 years of age has excellent short-term and intermediate-term outcome and is comparable to those aged 85 and younger. This study underlines the important role of the Heart Team in selecting patients eligible for TAVR and thus ensuring treatment success. Further research is needed to establish which of the patients aged over 85 benefit the most from this treatment.
